# Research on Packaging Reliability and Quality Factor Degradation Model for Wafer-Level Vacuum Sealing MEMS Gyroscopes

**DOI:** 10.3390/mi14101956

**Published:** 2023-10-20

**Authors:** Yingyu Xu, Shuibin Liu, Chunhua He, Heng Wu, Lianglun Cheng, Qinwen Huang, Guizhen Yan

**Affiliations:** 1 School of Computer, Guangdong University of Technology, Guangzhou 510006, China; 2 Science and Technology on Reliability Physics and Application Technology of Electronic Component Laboratory, China Electronic Product Reliability and Environmental Testing Research Institute, Guangzhou 510006, China; 3 National Key Laboratory of Science and Technology on Micro/Nano Fabrication, Institute of Microelectronics, Peking University, Beijing 100871, China

**Keywords:** MEMS gyroscope, quality factor, packaging reliability, wafer-level vacuum sealing, degradation model

## Abstract

MEMS gyroscopes are widely applied in consumer electronics, aerospace, missile guidance, and other fields. Reliable packaging is the foundation for ensuring the survivability and performance of the sensor in harsh environments, but gas leakage models of wafer-level MEMS gyroscopes are rarely reported. This paper proposes a gas leakage model for evaluating the packaging reliability of wafer-level MEMS gyroscopes. Based on thermodynamics and hydromechanics, the relationships between the quality factor, gas molecule number, and a quality factor degradation model are derived. The mechanism of the effect of gas leakage on the quality factor is explored at wafer-level packaging. The experimental results show that the reciprocal of the quality factor is exponentially related to gas leakage time, which is in accordance with the theoretical analysis. The coefficients of determination (*R*^2^) are all greater than 0.95 by fitting the curves in Matlab R2022b. The stable values of the quality factor for drive mode and sense mode are predicted to be 6609.4 and 1205.1, respectively, and the average degradation characteristic time is 435.84 h. The gas leakage time is at least eight times the average characteristic time, namely 3486.72 h, before a stable condition is achieved in the packaging chamber of the MEMS gyroscopes.

## 1. Introduction

The microelectromechanical system (MEMS) represents a sophisticated microintelligent system that leverages microelectronics, micromechanics, and related technologies to integrate sensors, actuators, and signal transmission components [1]. MEMS sensors exhibit key attributes such as compact size, lightweight design, and cost-effectiveness, and they are on the brink of achieving passive operation, miniaturization, and immunity to interference [2]. With the relentless progression of technology, MEMS sensors have found widespread utility across various domains, including consumer electronics, aerospace, military equipment, biomedicine, and more [3]. Notably, MEMS sensors usually rely on movable resonant structures, such as gyroscopes and accelerometers.

To optimize the performance of these devices, it is important to reduce the air damping of their moving structures. High-vacuum packaging serves to achieve this point, thereby obtaining a high Q-value, particularly in the case of gyroscopes [4]. Nowadays, the Q-value of high-performance gyroscopes can exceed 100,000 or even 1,000,000, underscoring the increasing importance of packaging reliability [5]. However, it is imperative to acknowledge that the vacuum within the MEMS chamber is subject to gradual degradation over time, exerting a direct impact on the performance of vacuum-packaged MEMS sensors and posing a significant threat to their long-term reliability [6]. Consequently, the issues of vacuum degradation and subsequent failure represent recurrent challenges encountered in the context of vacuum-packaged MEMS sensors [7].

Wafer-level packaging encapsulates MEMS structures on silicon chips between bonded wafers through surface micromachining and electrical interconnections [8,9]. It has the advantages of low cost, mass production, and high reliability. In 2019, Faisal Iqbal et al. fabricated a multiaxis gyroscope at 100 mTorr using wafer-level packaging, which has a high sensitivity of 12.56 μV/dps, 17.13 μV/dps, and 25.79 μV/dps in the roll, pitch, and yaw modes, respectively [10]. In 2021, Mustafa Mert Torunbalci et al. proposed a simple all-silicon wafer-level packaging method that achieves good hermeticity after temperature cycling (25–85 °C) and harsh temperature shocks (5 min @ 300 °C) [11]. A MEMS disk resonant gyroscope based on wafer-level packaging was reported by Hao Wang et al., achieving an angular random walk (ARW) of 0.05${}^{\circ}/\sqrt{h}$ and a bias instability of 0.42°/h within a full scale of ±300°/s [12].

While previous studies have established the relationship between the quality factor and temperature [13,14,15,16], research on the relationship between the quality factor and air pressure has rarely been reported. It is noteworthy that the impact of temperature on sensors can be mitigated through circuit compensation [17,18], but the effect of variations in gas pressure is generally difficult to compensate by circuitry, which leads to significant degradation of gyroscope performance [19,20]. Therefore, this paper focuses on elucidating the effects of pressure changes on the Q-value of MEMS gyroscopes.

Apart from temperature, gas leakage will cause pressure fluctuations in MEMS gyroscopes [21,22]. The internal pressure and leakage rate of the wafer-level packaged gyroscopes can be monitored by FIB and capacitance [23,24]. In 2019, Hengmao Liang et al. proposed a low-cost, 3D wafer-level packaging technology based on a coplanar Au-Si bonding structure [25]. The leak rate of the packaged chips (the cavity volume is ~0.0015 cc) is 5 × 10^−8^ atm·cc s^−1^ according to the MIL-STD-883F standard [26]. A wafer-level vacuum encapsulation method for sealing cavities by Au-Al thermocompression bonding at 250 °C has been proposed with a leak rate smaller than 2.8 × 10^−14^ mbarL/s [27]. In 2021, a novel solid–liquid interdiffusion (SLID) bonding process was reported, which yielded a leak value lower than 0.1 × 10^−9^ atm·cm^3^/s [28]. Nevertheless, there is a lack of theoretical studies on the model of gas leakage within wafer-level packaged gyroscopes. As a result, analyzing the pressure variation caused by gas leakage at wafer-level packaging is the focus of this work.

To eliminate the effect of temperature on pressure, it is essential to develop a degradation model at room temperature [29]. In our previous work, we developed a quality factor degradation model for device-level packaged gyros based on internal material outgassing, ignoring the effect of air leakage [30]. Considering that wafer-level packaging is an inevitable trend for high-performance gyroscopes, we performed long-term degradation tests for the two-mode quality factors of vacuum-sealed gyroscopes stored at room temperature, aiming to investigate packaging reliability and the degradation model for quality factors in wafer-level packaged gyroscopes.

## 2. Theoretical Analyses

**Figure 1:**
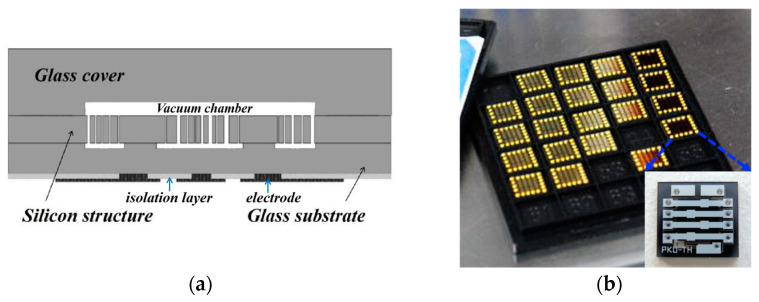
(**a**) Schematic of a wafer-level vacuum-packaged gyroscope. (**b**) Physical diagram of wafer-level vacuum-packaged gyroscopes.

The wafer-level vacuum-packaged gyroscope is shown in Figure 1. The movable structure of the gyroscope is enclosed within a vacuum chamber, utilizing a “sandwich” structure by anodic bonding between the glass substrate and the glass cover. The glass base below contains the electrodes and isolation layers. However, gas leakage within the encapsulation leads to an increase in the number of free gas molecules, resulting in variations in internal pressure and quality factors in the cavity.

**Figure 2:**
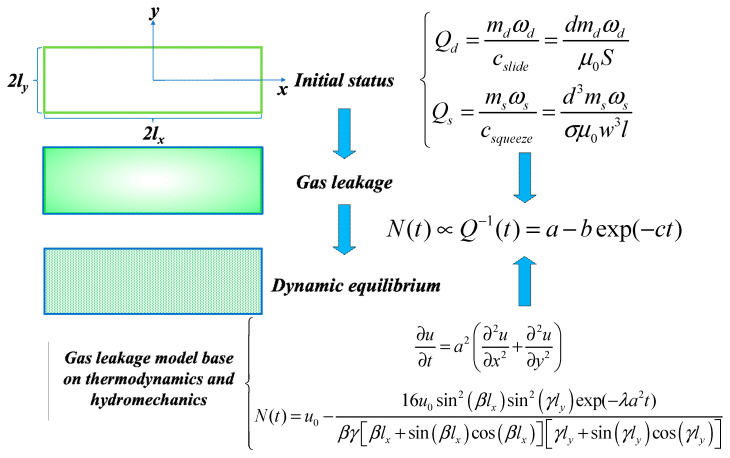
The simplified process of gas leakage, model derivation of quality factors (*Q_d_* and *Q_s_*), and number of gas molecules (*N*).

Figure 2 illustrates a simplified gas leakage process in the vacuum chamber. As the length and width of the vacuum-encapsulated cavity are significantly larger than the height, the gas diffusion region is seen as a two-dimensional plane. Assuming that initially there is a vacuum in the cavity, the outside gas diffuses from the boundary to the center of the cavity. The gas molecules are uniformly distributed along the x-z cross-section and the y-z cross-section, with diffusion along the x and y axes. Eventually, the gas within the cavity will reach dynamic equilibrium with the external gas at room temperature. Therefore, based on the principles of thermodynamics and fluid mechanics, the relationships between the quality factors of dive mode and sense mode (*Q_d_* and *Q_s_*), pressure (*p*), gas molecule number (*N*), and their degradation models can be derived in detail.

### 2.1. The Effect Mechanism of Gas Leakage

The relationship between the quality factor, air pressure, and gas number can be deduced as follows:

According to the principle of thermodynamics, the gas viscosity coefficient *μ_0_* can be obtained as [31]:

(1) $$\mu_{0}=\frac{1}{3}\rho \bar{v}\bar{\lambda }=\frac{N\bar{v}\bar{\tau }}{3V}\sqrt{\frac{8k_{b}Tm_{0}}{\pi }}$$

where $\bar{v}$ is mean velocity, $\bar{\lambda }$ is mean free path, and ρ is gas density, which equations can be expressed from Maxwell’s velocity distribution law; *k_b_*, *m*_0_, and $\bar{\tau }$ are the Boltzmann constant, the mass of a molecule, and the mean free time, respectively; *T* is the temperature; *V* is the volume of the sealed cavity; and *N* is the number of gas molecules.

The quality factor is strongly related to the air damping of the gyroscope, which mainly includes slide-film damping and squeeze-film damping. Slide-film damping is caused by the parallel motion of two flat plates while maintaining a constant gap. Squeeze-film damping is caused by the relative vertical movement of the two flat plates. Based on thermodynamic theory, the damping coefficient of slip-film combs $c_{slide}$ and squeeze-film combs $c_{squeeze}$ can be expressed as [32]:

(2) $$\left\{\begin{array}{l} c_{slide}=\frac{\mu_{0}S}{d}\\ c_{squeeze}=\frac{\sigma \mu_{0}w^{3}l}{d^{3}} \end{array}\right.$$

where *S* is the overlapped area of the plate capacitor, *d* is the gap between the combs, *w* and *l* are the width and length of the plate capacitor, respectively, and σ is a parameter determined by w/l. If w/l=1, then σ=0.427. If w/l→0, then σ=1. Then, we can obtain [33]:

(3) $$\frac{c_{squeeze}}{c_{slide}}=\frac{\sigma w^{3}l}{d^{2}S}=\frac{\sigma w^{2}}{d^{2}}$$

Based on the definition of the quality factor, the quality factors of the two modes can be derived as [33]:

(4) $$Q_{s}=\frac{m_{s}\omega_{s}}{c_{squeeze}}=\frac{d^{3}m_{s}\omega_{s}}{\sigma \mu_{0}w^{3}l},\;Q_{d}=\frac{m_{d}\omega_{d}}{c_{slide}}=\frac{dm_{d}\omega_{d}}{\mu_{0}S}$$

where *m_s_*, *c_s_*, $\omega_{s}$, and *Q_s_* represent the mass, damping coefficient, resonant frequency, and quality factor of the sense mode, respectively. *m_d_*, *c_d_*, $\omega_{d}$, and *Q_d_* represent the mass, damping coefficient, resonant frequency, and quality factor of the drive mode, respectively.

According to the ideal gas state equation, there is [31]:

(5) $$pV=Nk_{b}T$$

where *p* is the internal pressure. Thus, utilizing the previous equation, we can deduce [30]:

(6) $$Q_{s}=\frac{3\pi d^{3}m_{s}\omega_{s}}{8\sigma w^{3}l\bar{\tau }p},\;Q_{d}=\frac{3\pi dm_{d}\omega_{d}}{8S\bar{\tau }p}$$

The relationships between the quality factor, air pressure, and gas number can be simplified to

(7) $$p(t)\propto N(t)\propto Q^{-1}(t)$$

It can be seen that the internal pressure is directly proportional to the number of gas molecules and inversely proportional to the quality factor. Therefore, gas leakage due to the failure of the wafer package will increase the internal air pressure, resulting in a decrease in the quality factor.

### 2.2. Degradation Models Based on Gas Leakage

Simplify the enclosed cavity into a rectangle with length and width of 2*l_x_* and 2*l_y_*, respectively. By assuming that the cavity is initially under vacuum, it is inferred that external gas molecules will gradually diffuse from the boundary toward the center. Taking the center of the cavity as the origin, establish a rectangular coordinate system, as shown in the figure. Due to the symmetry of gas diffusion, only the first quadrant is analyzed here. The maximum diffusion lengths along the x and y directions are *l_x_* and *l_y_*, respectively. The gas diffusion equation and boundary conditions are as follows:

(8) $$\left\{\begin{array}{ll} \frac{\partial u}{\partial t}=D\left(\frac{\partial^{2}u}{\partial x^{2}}+\frac{\partial^{2}u}{\partial y^{2}}\right), & \left(0<x\leq l_{x},0<y\leq l_{y}\right)\\ \frac{\partial u}{\partial x}\left|{}_{x=0}\right.=\frac{\partial u}{\partial y}\left|{}_{y=0}\right.=0, & \frac{\partial u}{\partial x}\left|{}_{x=l_{x}}\right.=\frac{\partial u}{\partial y}\left|{}_{y=l_{y}}\right.=-\frac{b}{k}(u-u_{0})\\ u\left(x,y,0\right)=0 & \end{array}\right.$$

where u(x,y,t) is the instantaneous gas density, *x* and *y* are the distances from the center of the cavity, respectively, *t* is the gas diffusion time, *u*_0_ represents the external gas density, which affects the rate of gas leakage, *D* is the gas diffusion coefficient, and *b* and *k* represent the heat transfer coefficient and thermal conductivity, respectively.

If $\theta =u-u_{0}$, the equation for the definite solution is:

(9) $$\left\{\begin{array}{ll} \frac{\partial \theta }{\partial t}=D\left(\frac{\partial^{2}\theta }{\partial x^{2}}+\frac{\partial^{2}\theta }{\partial y^{2}}\right), & \left(0<x\leq l_{x},0<y\leq l_{y}\right)\\ \frac{\partial \theta }{\partial x}\left|{}_{x=0}\right.=\frac{\partial \theta }{\partial y}\left|{}_{y=0}\right.=0, & \frac{\partial \theta }{\partial x}\left|{}_{x=l_{x}}\right.=\frac{\partial \theta }{\partial y}\left|{}_{y=l_{y}}\right.=-\frac{b}{k}\theta \\ \theta \left(x,y,0\right)=-u_{0}=\theta_{0} & \end{array}\right.$$

First, we assume that θ(x,y,t)=X(x)Y(y)T(t) by using the method of separation of variables, and we can obtain:

(10) $$X(x)Y(y)\frac{dT}{dt}=D(Y(y)T(t)\frac{\partial^{2}X}{\partial x^{2}}+X(x)T(t)\frac{\partial^{2}Y}{\partial y^{2}})$$

Equation (10) can be expressed as:

(11) $$\frac{1}{DT(t)}\frac{dT}{dt}=\frac{1}{X(x)}\frac{\partial^{2}X}{\partial x^{2}}+\frac{1}{Y(y)}\frac{\partial^{2}Y}{\partial y^{2}}=-\lambda$$

If both sides of Equation (11) are equal to the constant −*λ*, we can obtain:

(12) $$T^{\prime }(t)+\lambda DT(t)=0$$

(13) $$\frac{1}{X(x)}\frac{\partial^{2}X}{\partial x^{2}}=-\left(\lambda +\frac{1}{Y(y)}\frac{\partial^{2}Y}{\partial y^{2}}\right)=-\mu$$

Similarly, if both sides of Equation (13) are equal to a constant −μ, the equation for *X(x)* can be isolated as follows:

(14) $$\left\{\begin{array}{c} X^{\prime \prime }(x)+\mu X(x)=0\\ X^{\prime }(0)=0,X^{\prime }(l_{x})=-\frac{b}{k}X \end{array}\right.$$

First, we discuss the solution to Equation (14).

① If μ<0, assuming that $\mu ={-\beta }^{2}$ and β>0, then the general solution of Equation (14) is:

(15) $$X(x)=C_{1}e^{\beta x}+C_{2}e^{-\beta x}$$

From boundary condition $X^{\prime }(0)=0$, we can obtain $X^{\prime }(0)=(C_{1}-C_{2})\beta =0$. Since β>0, $C_{1}=C_{2}$. From boundary condition $X^{\prime }(l_{x})=-\frac{b}{k}X$, we can yield:

(16) $$C_{1}\beta (e^{\beta l_{x}}-e^{-\beta l_{x}})=-\frac{b}{k}(e^{\beta l_{x}}+e^{-\beta l_{x}})C_{1}$$

If $C_{1}\neq 0$, then Equation (16) can be shown as:

(17) $$-\frac{k}{bl_{x}}\beta l_{x}=\frac{e^{\beta l_{x}}+e^{-\beta l_{x}}}{e^{\beta l_{x}}-e^{-\beta l_{x}}}=coth(\beta l_{x})$$

**Figure 3:**
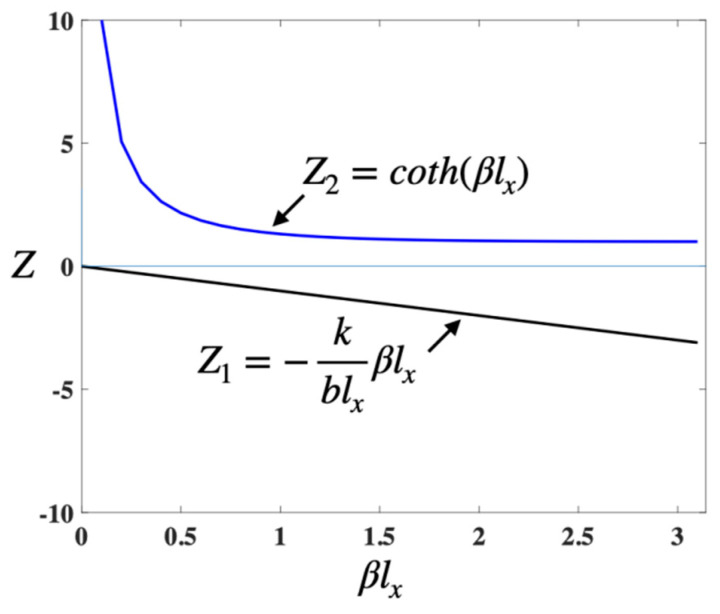
Diagram of the solution to Equation (17).

Now, assuming that $Z_{1}=-\frac{k}{bl_{x}}\beta l_{x}$ and $Z_{2}=coth(\beta l_{x})$, we found that $Z_{1}$ and $Z_{2}$ have no intersection from Figure 3. Equation (17) has solutions only if $C_{1}=0$. We can obtain $C_{1}=C_{2}=0$ and X(x)≡0, which is a trivial solution. Therefore, μ<0 is not valid.

② If μ=0, then the solution of Equation (14) is

(18) $$X(x)=C_{1}x+C_{2}$$

Substituting $X^{\prime }(0)=0$ into Equation (18), we can yield $C_{1}=0$. Substituting $X^{\prime }\left(l_{x}\right)=-\frac{b}{k}X$ into Equation (18), we can obtain $C_{1}=-\frac{b}{k}{(C}_{1}l_{x}+C_{2})$. Thus, $C_{2}=0$, X(x)≡0, and μ=0 are not valid.

③ If μ>0, assuming that $\mu =\beta^{2}\;and\;\beta >0$, then the general solution of Equation (14) is

(19) $$X(x)=C_{1}\sin\beta x+C_{2}\cos\beta x$$

We can obtain $C_{1}=0$ by substituting $X^{\prime }(0)=0$ into Equation (19). Equation (14) is derived as

(20) $$X(x)=C_{2}\cos\beta x$$

Equation (13) can be deduced as

(21) $$\frac{1}{Y(y)}\frac{\partial^{2}Y}{\partial y^{2}}=-\lambda +\mu$$

If λ=μ+η, then the equation for *Y*(*y*) is obtained as

(22) $$\left\{\begin{array}{c} Y^{\prime \prime }(y)+\eta Y(y)=0\\ Y^{\prime }(0)=0,Y^{\prime }(l_{y})=-\frac{b}{k}Y \end{array}\right.$$

Similarly, Equation (22) has a nontrivial solution only if η>0. Supposing $\eta =\gamma^{2}$, the general solution of Equation (15) is $Y(y)=D_{1}sin(\gamma y)+D_{2}cos(\gamma y)$. We can yield $D_{1}=0$ by substituting the boundary condition into the above formula. Consequently, the solution to Equation (22) is:

(23) $$Y(y)=D_{2}\cos\gamma y$$

For the differential Equation (12), the categorical variables are used. Integrating both sides and taking the logarithm, it is obtained as:

(24) T(t)=Aexp(−λDt)

where *A* is the integration constant, and θ(x,y,t) can be written as

(25) $$\theta =A\exp(-\lambda Dt)C_{2}\cos(\beta x)D_{2}\cos(\gamma y)$$

Substituting Equations (20) and (23) into the boundary conditions $X^{\prime }\left(l_{x}\right)=-\frac{b}{k}X$ and $Y^{\prime }\left(l_{y}\right)=-\frac{b}{k}Y$, respectively, we can yield:

(26) $$\cot(\beta l_{x})=\frac{\beta k}{b}$$

(27) $$\cot(\gamma l_{y})=\frac{\gamma k}{b}$$

**Figure 4:**
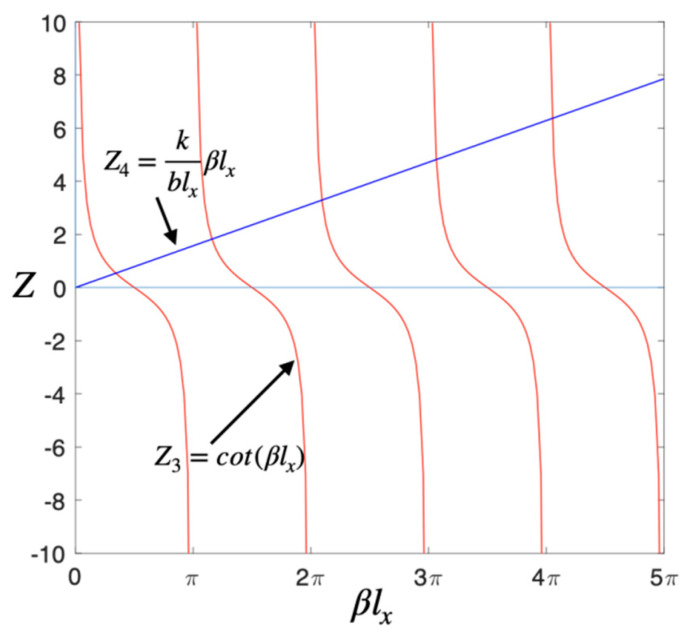
Diagram of the solution to the transcendental Equation (26).

The transcendental Equation (26) can be solved by the graphical method. Supposing that $Z_{3}=cot(\beta l_{x})$ and $Z_{4}=\frac{k}{bl_{x}}\beta l_{x}$, the curves for $Z_{3}$ and $Z_{4}$ are shown in Figure 4, respectively. The intersections of the curves are the solutions of Equation (26).

According to the above graph, there are infinite values of *β* (noted as $\beta_{1},\beta_{2}\ldots \ldots \beta_{m}$). Each *β* corresponds to one form of Equation (25). Similarly, we found numerous γ (noted as $\gamma_{1},\gamma_{2}\ldots \ldots \gamma_{n}$) based on the graphical method and corresponding Equation (23). By the superposition principle of solution, Equation (25) can be written as

(28) $$\theta (x,y,t)=\sum_{m=1}^{\infty }\sum_{n=1}^{\infty }\exp(-\lambda Dt)E_{1m}\cos(\beta_{m}x)G_{1n}\cos(\gamma_{n}y)$$

where E_1*m*_ and G_1*n*_ are integration constants, and *A* is combined with E_1*m*_. To solve for E_1*m*_ and G_1*n*_, substituting Equation (28) into the boundary condition $\theta (x,y,0)=-u_{0}=\theta_{0}$, we can yield:

(29) $$\theta_{0}=\sum_{m=1}^{\infty }\sum_{n=1}^{\infty }E_{1m}G_{1n}\cos(\beta_{m}x)\cos(\gamma_{n}y)$$

Equation (30) multiplied by $cos(\beta_{p}x)dx,\;cos(\gamma_{q}y)dy$ and integrated from −*l_x_* to *l_x_* for *x* and from −*l_y_* to *l_y_* for *y*, respectively. It can be deduced as:

(30) $$\theta_{0}\int_{-l_{x}}^{l_{x}}\int_{-l_{y}}^{l_{y}}\cos\beta_{p}x\cos\gamma_{q}ydxdy=\int_{-l_{x}}^{l_{x}}\int_{-l_{y}}^{l_{y}}\sum_{m=1}^{\infty }\sum_{n=1}^{\infty }E_{1m}G_{1n}\cos\beta_{m}x\cos\gamma_{n}y\cos\beta_{p}x\cos\gamma_{q}ydxdy$$

Since the above integrals are orthogonal, Equation (30) can be written as:

(31) $$\theta_{0}\int_{-l_{x}}^{l_{x}}\cos\beta_{p}xdx\int_{-l_{y}}^{l_{y}}\cos\gamma_{q}ydy=E_{1m}G_{1n}\int_{-l_{x}}^{l_{x}}\cos^{2}\left(\beta_{m}x\right)dx\int_{-l_{y}}^{l_{y}}\cos^{2}\left(\gamma_{n}y\right)dy$$

Therefore, the solution of E_1*m*_G_1*n*_ is deduced as:

(32) $$E_{1m}G_{1n}=\frac{\theta_{0}4\sin\left(\beta_{m}l_{x}\right)\sin\left(\gamma_{n}l_{y}\right)}{\left[\beta_{m}l_{x}+\sin\left(\beta_{m}l_{x}\right)\cos\left(\beta_{m}l_{x}\right)\right]\left[\gamma_{n}l_{y}+\sin\left(\gamma_{n}l_{y}\right)\cos\left(\gamma_{n}l_{y}\right)\right]}$$

Substitute Equation (32) into Equation (28). From Equation (9), we know that $\theta_{0}=-u_{0}$. Equation (28) can then be written as:

(33) $$\theta =-\sum_{m=1}^{\infty }\sum_{n=1}^{\infty }\frac{u_{0}4\sin\left(\beta_{m}l_{x}\right)\sin\left(\gamma_{n}l_{y}\right)\cos\beta_{m}x\cos\gamma_{n}y\exp(-\lambda_{mn}Dt)}{\left[\beta_{m}l_{x}+\sin\left(\beta_{m}l_{x}\right)\cos\left(\beta_{m}l_{x}\right)\right]\left[\gamma_{n}l_{y}+\sin\left(\gamma_{n}l_{y}\right)\cos\left(\gamma_{n}l_{y}\right)\right]}$$

The higher-order terms are ignored in the above equations, only the first-order component is considered (*m* = 1, *n* = 1), and $\theta =u-u_{0}$. The gas density can be deduced as:

(34) $$u=u_{0}-\frac{u_{0}4\sin\left(\beta l_{x}\right)\sin\left(\gamma l_{y}\right)\cos\beta x\cos\gamma y\exp(-\lambda Dt)}{\left[\beta l_{x}+\sin\left(\beta l_{x}\right)\cos\left(\beta l_{x}\right)\right]\left[\gamma_{n}l_{y}+\sin\left(\gamma l_{y}\right)\cos\left(\gamma l_{y}\right)\right]}$$

Integrating θ(x,y,t) over −*l_x_* to *l_x_* and −*l_y_* to *l_y_*, the number of gas molecules *N*(*t*) in the cavity can be calculated as:

(35) $$N(t)=4l_{x}l_{y}u_{0}-\frac{16u_{0}\sin^{2}\left(\beta l_{x}\right)\sin^{2}\left(\gamma l_{y}\right)\exp(-\lambda Dt)}{\beta \gamma \left[\beta l_{x}+\sin\left(\beta l_{x}\right)\cos\left(\beta l_{x}\right)\right]\left[\gamma l_{y}+\sin\left(\gamma l_{y}\right)\cos\left(\gamma l_{y}\right)\right]}$$

Consequently, the degradation model can be obtained as

(36) $$Q^{-1}(t)\propto N(t)=a-b\exp(-ct)$$

where *a*, *b*, and *c* are constants, which can be yielded by comparing Equations (35) and (36).

(37) $$a=4l_{x}l_{y}u_{0}$$

(38) $$b=\frac{16u_{0}\sin^{2}\left(\beta l_{x}\right)\sin^{2}\left(\gamma l_{y}\right)}{\beta \gamma \left[\beta l_{x}+\sin\left(\beta l_{x}\right)\cos\left(\beta l_{x}\right)\right]\left[\gamma l_{y}+\sin\left(\gamma l_{y}\right)\cos\left(\gamma l_{y}\right)\right]}$$

(39) c=λD

The coefficients *a*, *b*, and *c* depend on factors such as the dimensions of the vacuum chamber, external temperature, pressure, etc. The physical meanings of the model parameters *a*, *b*, and *c* are as follows:

Parameter *a* denotes the quantity of gas molecules in the sample cavity, determined by the cavity size and external gas density. It also governs the final quality factor’s magnitude. Parameter *b* indicates that chamber size and pressure affect the degradation level. Parameter *c* characterizes the quality factor’s degradation rate, which is dependent on the gas diffusion coefficient and temperature.

Since equation ${Q(t)}^{-1}\propto N(t)=a-bexp(-ct)$ serves as a simplified model, Equations (37)–(39) can only calculate the parameters *a*, *b*, and *c* in equation $N\left(t\right)=a-bexp(-ct)$, which are used to predict the number of gas molecules *N*(*t*). The parameters *a*, *b*, and *c* fitted from the experimental data are specific to equation ${Q(t)}^{-1}=a-bexp(-ct)$, which is used to predict the quality factor *Q*(*t*). In summary, while the parameters in equation $N\left(t\right)=a-bexp(-ct)$ can be computed using Equations (37)–(39), the parameters in equation ${Q(t)}^{-1}=a-bexp(-ct)$ cannot be directly obtained by Equations (37)–(39).

When t=0, N(t)≈0, which means that the number of gas molecules in the initial state of the chamber is approximately 0, namely, the vacuum state. The correctness of the theoretical derivation is confirmed by fitting the experimental data in Matlab to obtain *a* ≈ *b*. When *t* tends to infinity, $lim_{t\to \infty }N\left(t\right)=4l_{x}l_{y}u_{0}$, which means that the number of gas molecules tends to a constant value $4l_{x}l_{y}u_{0}$, reaching the equilibrium state. The number of gas molecules in the cavity is predicted by experimentally measuring the external gas density $u_{0}$. At the same time, the Q-value of both drive and sense modes reach stable values. We, therefore, define a constant $k_{q}$ to describe the magnitude of the change of the Q-value. The quality factor can be considered to reach a stable value when $k_{q}=\frac{\left|∆Q\right|}{Q}<1\%$.

## 3. Experimental Results

To research the degradation of gyroscopes due to gas leakage in a practical situation, a long-term degradation test of the two-mode quality factor was performed over 8 months. During this period, the gyroscope was stored in a temperature-controlled chamber at room temperature (25 °C). The two-mode quality factors corresponding to different times were obtained through several tests. The results indicate a decrease in the quality factors from several hundred thousand to several thousand. The internal encapsulation leaks slowly at room temperature, and the air leakage causes a rise in air pressure and a decrease in quality factors. It takes about 4 months to enter a plateau of natural degradation, but it is not the end state. The relationship between 1/*Q_d_* and air leakage time *t* is approximately exponential, as is the relationship between 1/*Q_s_* and gas leakage time *t*, according to the data fitting analysis using Matlab. It is consistent with the analytical model of Equation (36), thus validating the correctness of the theoretical analysis.

**Figure 5:**
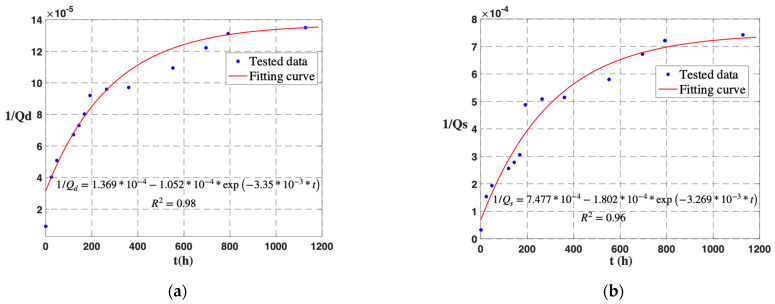
During approximately 1.5 months, the reciprocal of the quality factor is exponentially related to the gas leakage time. (**a**) Drive mode. (**b**) Sense mode.

During a natural degradation time of about 1.5 months, it can be concluded that parameters *a*, *b*, and *c* of the drive mode are 1.369 ∗ 10^−4^, 1.052 ∗ 10^−4^, and 3.35 ∗ 10^−3^, respectively. The fitted curve is $1/Q_{d}=1.369*10^{-4}-1.052*10^{-4}*\exp(-3.35*10^{-3}*t)$. The coefficient of determination (*R*^2^) is 0.98. For the sense mode, parameters *a*, *b*, and *c* are 7.477 ∗ 10^−4^, 1.802 ∗ 10^−4^, and 3.269 ∗ 10^−3^, respectively. The fitted equation is $1/Q_{s}=7.477*10^{-4}-1.802*10^{-4}*\exp(-3.269*10^{-3}*t)$. The coefficient of determination (*R*^2^) is 0.96. The test results and fitting curves are shown in Figure 5.

**Figure 6:**
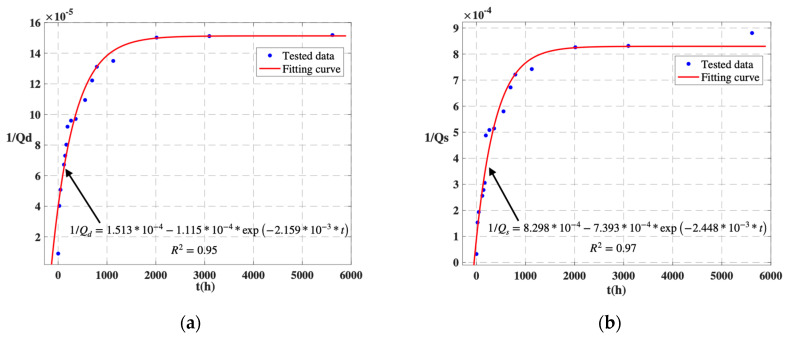
During approximately 8 months, the reciprocal of the quality factor is exponentially related to the gas leakage time. (**a**) Drive mode. (**b**) Sense mode.

Over a period of approximately 8 months of natural degradation, the parameters *a*, *b*, and *c* of the drive mode are 1.513 ∗ 10^−4^, 1.115 ∗ 10^−4^, and 2.159 ∗ 10^−3^, respectively. The fitted equation is $1/Q_{d}=1.513*10^{-4}-1.115*10^{-4}*\exp(-2.159*10^{-3}*t)$. The coefficient of determination (*R*^2^) is 0.95. For the sense mode, parameters *a*, *b*, and *c* are 8.298 ∗ 10^−4^, 7.393 ∗ 10^−4^, and 2.448 ∗ 10^−3^, respectively. The fitted equation is $1/Q_{s}=8.298*10^{-4}-7.393*10^{-4}*\exp(-2.448*10^{-3}*t)$. The coefficient of determination (*R*^2^) is 0.97. The test results and fitting curves are shown in Figure 6. Thus, for *Q_d_* and *Q_s_*, the final values of 1/a at room temperature are 6609.4 and 1205.1, respectively.

In addition, the fitted values of 1.369 ∗ 10^−4^ and 1.513 ∗ 10^−4^ for the drive mode do not differ greatly between two different time periods, and this is also the case for the sense mode. However, due to the differences in parameters such as mass and resonant frequency between the drive mode and the sense mode, the quality factors of the two modes are not the same. Consequently, this leads to variations in the fitting values of the two modes. It is important to note that ${Q(t)}^{-1}$ is proportional to N(t) rather than ${Q(t)}^{-1}$ being equal to N(t). According to the definition of characteristic time (i.e., the time corresponding to the completion of $e^{-1}$ of the air leakage process), it can be calculated that the characteristic times 1/c of drive mode and sense mode are 463.18 h and 408.5 h, respectively, and the average degradation characteristic time is 435.84 h.

The fitted curves in Figure 6 indicate that the curves change from a sharp rise to a slow rise at approximately 2000 h. After 2000 h, although the Q-value remains in a slow decline for a long period of time, $k_{q}$ is extremely small, and the Q-value is approximately stable. Based on the experimental data, the $k_{q}$ values for both the drive and detection modes are 0.007 at t=3096 h, indicating that the Q-value changes are extremely small and stable. Hence, to reach a steady state with air leakage inside the gyro package, the gas leakage time is at least eight times the average characteristic time, namely 3486.72 h. Since the natural aging time is too long for reliability design, temperature cycling tests for MEMS gyroscopes can be adopted to accelerate the encapsulation leakage process, thus improving the efficiency of package reliability research. Continuing this line of investigation will be a key focus of our future research.

## 4. Conclusions

This paper presents a novel method for assessing the reliability of wafer-level, packaged MEMS gyroscopes. Based on the principles of thermodynamics and hydrodynamics, a detailed derivation of the relationships between quality factor, air pressure, and gas number, as well as their degradation models, are presented. The results of degradation tests have demonstrated an exponential relationship between the reciprocal of the quality factor and the air leakage time at room temperature, which is consistent with the theoretical analysis, and the coefficients of determination (*R*^2^) are all greater than 0.95. Based on the experimental data, an equation describing the variation of quality factors *Q*(*t*) with leakage time *t* was obtained by Matlab. This equation makes it possible to predict the steady-state value of the quality factor under this condition, thereby assessing the reliability of the gyroscope sealing against gas leakage. The limit values of the quality factor for the two modes are predicted to be 6609.4 and 1205.1, respectively. The proposed model holds potential for implementation in reliability design and the establishment of standards for degradation tests.

By fitting the experimental data to a mathematical model, a quantitative relationship between the quality factor and time is established to extrapolate the long-term behavior of the gyroscope’s quality factor. This predictive capability provides valuable insight into the reliability of the gyroscope’s sealing mechanism, facilitating an evaluation of the stability and effectiveness of the encapsulation process in preventing gas leakage. However, it is important to note that gas leakage and natural degradation of the encapsulation at room temperature exhibit an extremely slow progression, taking more than 4 months to reach a degradation plateau. To expedite the design cycle for gas leakage package reliability, our future research will focus on temperature cycling tests of wafer-level gyroscopes to accelerate the gas leakage process. Additionally, these temperature cycling tests will foster the expansion of cracks at material interfaces, ultimately allowing leakage to reach a stable value. By reaching a steady state for gas leaks, the reliability of the gyroscope is expected to be notably enhanced.

In summary, this study presents a comprehensive approach to assessing the reliability of wafer-level packaged MEMS gyroscopes. The derived models and experimentally validated results provide a deeper understanding of the degradation behavior and sealing effectiveness of gas leakage. The proposed model has significant implications for reliability design and the establishment of degradation test standards in the field of MEMS gyroscopes.

## Data Availability

Not applicable.
